# P-1212. Enterovirus D68 (EV-D68) Incidence and Spectrum of Illness among Children 0-2 years in a U.S. Community-based Birth Cohort in Cincinnati, OH during 2017-2020

**DOI:** 10.1093/ofid/ofae631.1394

**Published:** 2025-01-29

**Authors:** Zheyi Teoh, Claire Midgley, Christina M Quigley, Shannon C Conrey, Allison R Cline, Amy Ostrow, Brendon White, Meredith L McMorrow, Daniel C Payne, Ardythe L Morrow, Mary A Staat

**Affiliations:** Seattle Children's Hospital, Seattle, Washington; Centers for Disease Control and Prevention, Atlanta, Georgia; Cincinnati Children's Hospital Medical Center, Cincinnati, Ohio; University of Cincinnati College of Medicine, Cincinnati, Ohio; University of Cincinnati College of Medicine, Cincinnati, Ohio; Cincinnati Children's Hospital Medical Center, Cincinnati, Ohio; Cincinnati Children's Hospital Medical Center, Cincinnati, Ohio; CDC/NCIRD/CORVD/SPB, Atlanta, GA; CDC, Decatur, Georgia; University of Cincinnati College of Medicine, Cincinnati, Ohio; Cincinnati Children’s Hospital Medical Center, Cincinnati, Ohio

## Abstract

**Background:**

The community burden and epidemiology of non-medically attended enterovirus-D68 (EV-D68) is unknown, as prior studies focus on EV-D68-associated asthma exacerbation, pneumonia, or acute flaccid myelitis (AFM) which occurs biennially. We describe EV-D68 incidence and spectrum of illness among children 0-2 years in Cincinnati, OH during 2017-2020.

**Figure:**
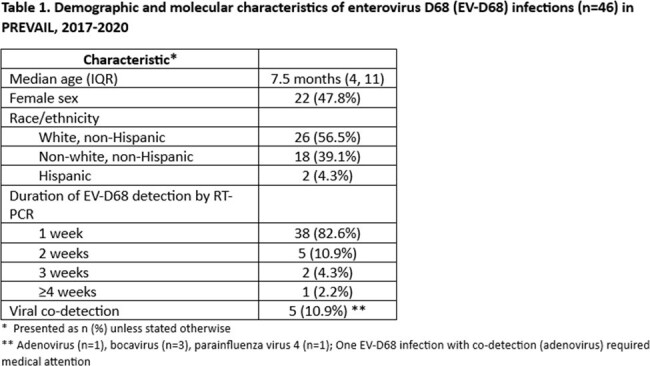

Demographic and molecular characteristics of enterovirus D68 (EV-D68) infections (n=46) in PREVAIL, 2017-2020

**Methods:**

The PREVAIL cohort is a community-based, longitudinal birth cohort of healthy mother-infant pairs with children observed from birth to 2 years of age. Nasal swabs were collected weekly from children and tested using the PCR-based Luminex Respiratory Pathogen Panel. Swabs positive for rhinovirus/enterovirus (RV/EV) between July-December, during which the typical U.S. enterovirus season occurs, were then tested for EV-D68 by RT-PCR. Weekly text surveys and chart abstraction identified symptoms and medically attended visits. Acute respiratory illness (ARI) was defined as the presence of cough or fever within 7 days of virus detection.

**Figure:**
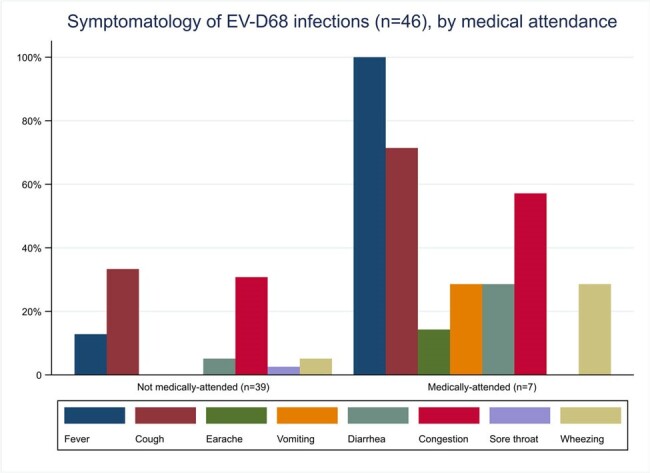

Symptomatology of enterovirus D68 (EV-D68) infections (n=46), by medical attendance

**Results:**

The PREVAIL Cohort enrolled 245 mother-infant pairs who submitted 13,781 infant nasal swabs during 04/2017-07/2020. A total of 1,424 swabs positive for RV/EV between July-December were further tested for EV-D68. Forty-six EV-D68 infections were detected, all occurring between 08/2018-11/2018; no detections occurred in 2017, 2019, or 2020. Estimated annual incidence of EV-D68 was 0.78/100 child-weeks (95%CI: 0.57-1.04) for 2018. Median age at EV-D68 infection was 7.5 months (Table 1). Of the 46 infections, 21 (46%) met ARI criteria; the remaining 25 (54%) were considered asymptomatic. Overall, cough (39%), nasal congestion (35%) or fever (26%) were the most prevalent symptoms, while diarrhea (9%) or vomiting (4%) were infrequent (Figure 1). Seven (15%) EV-D68 infections were medically attended, with 3 evaluated in an emergency department. No hospitalizations and no cases of pneumonia or AFM occurred.

**Conclusion:**

In our community cohort of healthy children aged < 2 years, less than half of EV-D68 infections met ARI criteria (46%), and 15% were medically attended; none required hospitalization. Prospective multi-year cohort studies in all ages would be valuable for understanding the full spectrum of symptoms, and burden of EV-D68.

**Disclosures:**

**Mary A. Staat, MD, MPH**, Cepheid: Grant/Research Support|Merck: Grant/Research Support|Pfizer: Grant/Research Support|Up-To-Date: Honoraria

